# Early, Chronic, and Acute Cannabis Exposure and Their Relationship With Cognitive and Behavioral Harms

**DOI:** 10.3389/fpsyt.2021.643556

**Published:** 2021-08-09

**Authors:** Hugo López-Pelayo, Eugènia Campeny, Clara Oliveras, Jürgen Rehm, Jakob Manthey, Antoni Gual, Maria de las Mercedes Balcells-Olivero

**Affiliations:** ^1^Grup Recerca en Addiccions Clinic (GRAC), Institut de Recerca Biomèdica August Pi i Sunyer (IDIBAPS), Barcelona, Spain; ^2^Psychiatry Department, Neuroscience Institute, Hospital Clínic de Barcelona, Barcelona, Spain; ^3^Campbell Family Mental Health Research Institute, Centre for Addiction and Mental Health, Toronto, ON, Canada; ^4^Department of Psychiatry, Dalla Lana School of Public Health, University of Toronto (UofT), Toronto, ON, Canada; ^5^Institute of Clinical Psychology and Psychotherapy, Technische Universität Dresden, Dresden, Germany; ^6^Department of International Health Projects, Institute for Leadership and Health Management, I.M. Sechenov First Moscow State Medical University, Moscow, Russia; ^7^Faculty of Psychology, Institute of Clinical Psychology and Psychotherapy, Technical University Dresden, Dresden, Germany; ^8^Department of Psychiatry and Psychotherapy, Center for Interdisciplinary Addiction Research of Hamburg University (ZIS), University Medical Center Hamburg-Eppendorf (UKE), Hamburg, Germany; ^9^Department of Psychiatry, Medical Faculty, University of Leipzig, Leipzig, Germany

**Keywords:** Cannabis, cognition, behavior, health, harm, THC, risk

## Abstract

**Background:** Cannabis is the third most consumed drug worldwide. Thus, healthcare providers should be able to identify users who are in need for an intervention. This study aims to explore the relationship of acute, chronic, and early exposure (AE, CE, and EE) to cannabis with cognitive and behavioral harms (CBH), as a first step toward defining risky cannabis use criteria.

**Methods:** Adults living in Spain who used cannabis at least once during the last year answered an online survey about cannabis use and health-related harms. Cannabis use was assessed in five dimensions: quantity on use days during the last 30 days (AE), frequency of use in the last month (AE), years of regular use (YRCU) (CE), age of first use (AOf) (EE), and age of onset of regular use (AOr) (EE). CBH indicators included validated instruments and custom-made items. Pearson correlations were calculated for continuous variables, and Student's *t*-tests for independent samples were calculated for categorical variables. Effect sizes were calculated for each of the five dimensions of use (Cohen's *d* or *r* Pearson correlation) and harm outcome. Classification and Regression Trees (CART) analyses were performed for those dependent variables (harms) significantly associated with at least two dimensions of cannabis use patterns. Lastly, logistic binary analyses were conducted for each harm outcome.

**Results:** The mean age of participants was 26.2 years old [standard deviation (SD) 8.5]. Out of 2,124 respondents, 1,606 (75.6%) reported at least one harm outcome (mean 1.8 and SD 1.5). In our sample, using cannabis on 3 out of 4 days was associated with an 8-fold probability of scoring 4+ on the Severity Dependence Scale (OR 8.33, 95% CI 4.91–14.16, *p* <0.001), which is indicative of a cannabis use disorder. Also, a start of regular cannabis use before the age of 25 combined with using cannabis at least once per month was associated with a higher probability of risky alcohol use (OR 1.33, 95% CI 1.12–1.57, *p* = 0.001). Besides, a start of regular cannabis use before the age of 18 combined with a period of regular use of at least 7.5 years was associated with a higher probability of reporting a motor vehicle accident (OR 1.81, 95% CI 1.41–2.32, *p* < 0.0001). Results were ambiguous regarding the role that age of first use and milligrams of THC per day of use might play regarding cannabis-related harms.

**Conclusions:** The relationship among AE, CE, and EE with CBH indicators is a complex phenomenon that deserves further studies. The pattern of cannabis use should be carefully and widely evaluated—(not just including frequency but also other dimensions of pattern of use)—in research (preferably in longitudinal studies) to assess cannabis-related harms.

## Background

Cannabis is the third most prevalent psychoactive substance used worldwide. Globally, there were an estimated 192 million past-year users of cannabis in 2018, corresponding to 3.9% of the global population aged 15–64 (1). In Europe, 90.2 million adults (aged 15–64), or 27.4% of this age group, reported lifetime cannabis use. Among this whole group, 7.6% reported use during the last year. The prevalence of last year use was higher (15.0%) among the younger ones (aged 15–34) (2).

Cannabis legislative frameworks are evolving worldwide (3, 4), and global tendencies point out that cannabis use is increasing, while the perception of risks associated to cannabis is declining (5). Previous literature has extensively documented multiple health-related harms associated with cannabis use. Besides several somatic harms such as respiratory adverse events, cancer, cardiovascular outcomes, and gastrointestinal disorders, the deleterious consequences of cannabis use on mental health, cognition, and behavior are well-documented (6, 7).

Regarding mental health, multiple studies have revealed a clear relationship between cannabis consumption, and both psychotic symptoms (8) and risk for developing schizophrenia, especially among heavy cannabis users, compared to non-users (9). Cannabis use has an impact on incidence of psychotic experiences (10). Moreover, age at onset of psychosis is on average 2.7 years earlier for cannabis users (11). Also, several studies have suggested that cannabis consumption may represent a risk factor for depression (12), mainly after long-term and heavy use (13). Cannabis use has also been associated with bipolar disorder (14) and the development of anxiety symptoms in the general population (15). Lastly, cannabis users may also develop a cannabis use disorder (16, 17). Using cannabis both daily and weekly, early onset of use (11–15 years) and the experience of positive psychotropic effects of cannabis are considered risk factors for onset of cannabis use disorders (18).

Additionally, there is enough evidence to endorse the claim of a negative impact of chronic cannabis use on cognition (19, 20), even after the person is no longer acutely intoxicated (“stoned”) by cannabis use. Memory is the most consistently impaired cognitive domain (21). Verbal learning and memory tasks seem to be distinctly sensitive to both the acute and chronic effects of cannabis, with mixed evidence regarding improvement with abstinence. Working memory seems to be affected by acute cannabis use and also by chronic use, mostly in young and adolescent users, but appears to mostly resolve with prolonged periods of abstinence. Although, impaired attention has often been considered an indication of the intoxicating effects of cannabis, there is evidence for both acute and chronic exposure impairing this cognitive domain. Psychomotor function is affected by acute intoxication and this likely persists for some time following chronic cannabis exposure. Regarding executive functions, there are clear acutely impairing effects on inhibition, whereas, planning, problem solving, reasoning and interference control may be more affected in older chronic users, or with greater exposure to cannabis. Risky decision making and sensitivity to reward are increased during acute intoxication but the extent to which these effects persist in chronic or abstinent users remains unclear (22). Chronic cannabis use also alters concentration (23).

Cannabis use also seems to be a risk factor for negative behavioral outcomes (24) such as suicidal behavior, violence, and motor vehicle accidents (25).

Up to this moment, there is insufficient evidence on what exactly risky use constitutes, making it difficult for healthcare providers to identify users who qualify for an intervention.

Previous studies usually focus on one single harm (e.g., anxiety) and one single dimension of pattern of cannabis use (e.g., frequency of use). Using the data obtained in a survey that was answered by a sample of 2,124 Spanish adult cannabis users, mostly men in their 20's with university degrees, we aim to explore the relationship among five dimensions of cannabis use (quantity, frequency, years of regular use, age of onset, and age of initiation regular use)—grouped as acute exposure (quantity and frequency of use), early exposure (age of onset and age of initiation of regular use), and chronic exposure (years of regular use)—and 12 indicators of cognitive and behavioral related harms.

Although the cross-sectional design of our study will not allow to establish causality or to assuredly define the cutoff point for frequency, quantity, age of first use, age of initiation of regular use, or years of regular use that affect harm, our results will increase the evidence in favor of considering not just frequency of use but also other dimensions of cannabis use in both research and clinical practice.

## Materials and Methods

### Design, Setting, and Procedure of the Study

**Figure 1:**
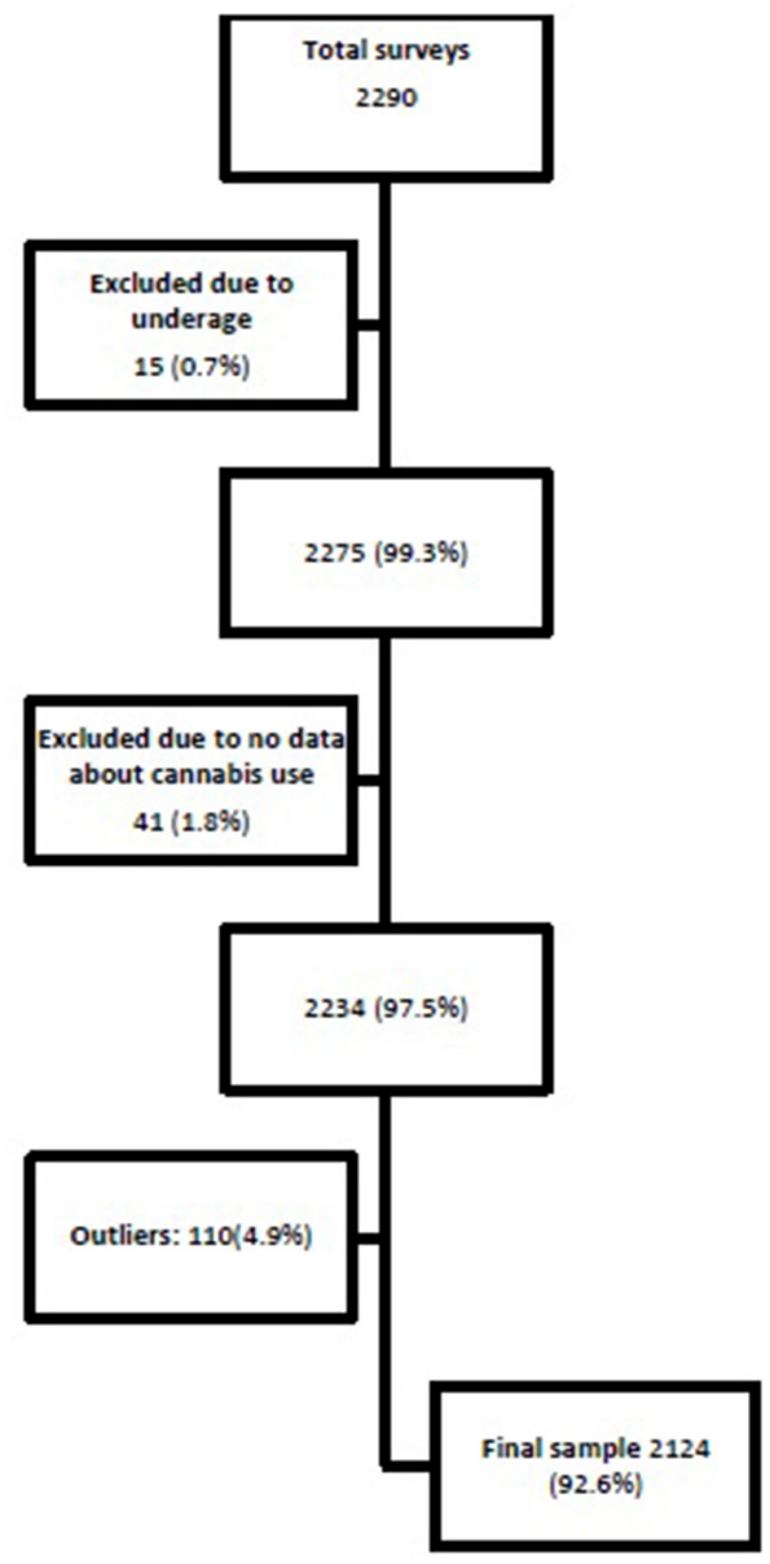
Flow process of sample selection.

The flow process is outlined in Figure 1. From March 2019 to February 2020, a sample of 2,124 people was recruited for a cross-sectional study. Adults (≥18 years old), living in Spain, who used cannabis at least once during the last 12 months were eligible to participate. Exclusion criteria were as follows: (a) no reported data about patterns of cannabis use; (b) idiomatic barriers (cannot understand Spanish); (c) incapacity to sign the informed consent; and (d) no access to the Internet. Outliers for two variables (milligrams THC per day of use during the last month and sum of harms) were excluded if *Z*-score ± 2.5 (deviation from mean).

An online survey was distributed among different organizations, which have access to people who use cannabis. They provided the link to access the survey about their internal networks. Five universities (including students associations), one federation of cannabis users association, seven media webs, eight researchers, and 13 other social and scientific organizations participated in the distribution.

The survey in itself was anonymous. However, upon completion of the survey, participants were given the opportunity to participate in a raffle of 10 vouchers for exchanging in a website of travels and gifts (138€ each voucher). The data collected as part of the raffle were not combined with survey responses at any time.

### Assessment

An online survey was designed based on a recent systematic review of systematic reviews about cannabis-related harm (7). The survey was tested through a pilot study (under review).

The survey included 55 questions (for more details, see Supplementary Materials), divided into four sections.

1) Socio-demographic characteristics (age, gender, education, marital status, and working status).
2) Substance use during the last 12 months (tobacco, alcohol, cocaine, opioids, amphetamines, LSD, and benzodiazepines without prescription); alcohol use was measured through the Alcohol Use Disorder Identification Test—C (AUDIT-C) (26).
3) Patterns of cannabis use, type of cannabis derivate use (herbal, hash, herbal and hash, other), administration route (smoked, ingested, vaping, other), frequency of use (number of days of use during the last month), age at first use and age of regular use onset (patients' self-perception), years of regular use (assessed according to years between age at regular use and current age), milligrams of delta-9-THC per day of use during the last month based on the Standard Joint Unit (quantity assessed according to the following equivalences: 7 mg delta-9-THC = 1 joint = 250 milligrams cannabis per joint = 1€) (27), and site of purchase (cannabis association, own production, dealer, friends, other or several ways).
4) Health status and injury background. The following scales were included in the survey for assessing psychological harms (found in the systematic review of the literature): (a) Severity Dependence Scale (SDS) for cannabis use (28); (b) General Anxiety Disorder-7 (GAD-7) (29); (c) Patient Health Questionnaire-9 (PHQ-9) (30); (d) Multicage CAD-4 for gaming (CAD-4) (31). Other harms were explored with (e) Sleep problems based on questions 2110–2111 of “World Health Organization Survey about health and health system responsiveness” (32) and (f) Cognitive impairment based on questions of the first domain (cognition) of “WHODAS 2.0. Measuring health and disability: manual for WHO disability assessment schedule” (33) that are also used in “World Health Organization Survey about health and health system responsiveness” (32): “In the past 30 days, how much difficulty did you have in: Concentrating on doing something for 10 min?, Remembering to do important things?, Analyzing and finding solutions to problems in day-to-day life?, Learning a new task, for example, learning how to get to a new place?”; (g) *Ad hoc* questions about violence: “Have you ever experienced any of the following situations in your family? (verbal violence and physical violence) Who perpetrated the violence?” (positive outcome was considered only if the user perpetrated physical violence); (h) *Ad hoc* questions about motor vehicle accidents: “Have you ever experienced a motor vehicle accident? Have you consumed cannabis the 6 h before the collision?” (positive outcome was considered only if positive answer to both questions) (7); (i) *Ad hoc* questions about mental health “Have you ever been diagnosed with any of the following illness? Depressive disorder; Anxiety disorder; Bipolar disorder; Other, specify; No, never”; (j) *Ad hoc* questions about suicidal impulses: “Have you ever thought of hurting yourself? Have you ever attempted?” (positive outcome was considered if affirmative response to at least one question); (k) *Ad hoc* questions about previous treatment for drug use disorders: “Have you ever been in treatment for any of the following substances? Alcohol; Cocaine; Cannabis; Heroin; Other, specify; No, never.”

### Statistical Analyses

A descriptive analysis of qualitative variables was conducted using frequencies and percentages. Mean and standard deviation (SD) were used for continuous variables.

#### Dependent Variables

Total score SDS, total score GAD-7, total score PHQ-9, total score CAD-4, and total score AUDIT-C. Two categories according to previous treatment for drug use disorder (no and yes), two categories for suicidal impulses (no and thoughts or attempts), two categories for mental health (no previous mental health diagnosis and previous mental health diagnosis), two categories for motor vehicle accidents (no or yes and cannabis use 6 h before collision), two categories for experience of violence (no and yes), two categories for cognitive impairment (no and yes), and two categories for sleep disorders (no and yes). Only for calculating the number of harms for each respondent was a cutoff established for SDS (>4), GAD7 (>4), PHQ-9 (>4), CAD-4 (>1), and AUDIT-C (>4).

#### Independent Variables

Milligrams THC per day of use during the last 30 days (measure of acute exposure), days of use during the last 30 days (DU) (measure of acute exposure), age of cannabis onset (AOf) (first use) (measure of early exposure), age of regular cannabis onset (AOr) (measure of early exposure), and years of regular use *(YRCU)* (measure of chronic exposure).

Univariate parametric tests were performed using Student's *t*-test for independent samples for categorical variables as independent and Pearson correlation tests for continuous variables. Standardized effect sizes (Cohen's *d*) were calculated for statistically significant associations (*p*-value < 0.05). For correlations, *r* < 0.10 was not interpreted, *r* between 0.10 and under 0.30 was considered a small effect size, *r* from 0.30 and under 0.50 was considered a medium effect size, and *r* value ≥ 0.50 was considered a large effect size. Cohen's *d* values were calculated; values under 0.20 were not interpreted, those between 0.20 and under 0.50 were considered small effect size, those between 0.50 and under 0.80 were considered medium, and those ≥0.80 were considered large effect size (34).

Since we aimed to explore the relationship between different dimensions of cannabis use patterns internally and with self-reported cannabis-related harms on a successive phase of exploration, we performed Classification and Regression Trees (CART) analyses for those dependent variables (harms) significantly associated with at least two dimensions of cannabis use patterns (independent). CART analyses are used as an exploration method to classify systems that differ due to natural causes, in our case patterns of cannabis use. CART analysis is a type of decision tree learning technique and, consequently, is useful for creating a predictive model. In our analyses, we used CART analyses to explore potential predictive models of cannabis-related harms based on patterns of cannabis use. As dependent variables, we included those harms with at least two dimensions of use patterns associated with a single harm in the univariate analyses, and as independent variables, we included those patterns of use associated with this specific variable. Based on the association of cannabis use patterns and experience of cannabis-related harms, the CART analysis allows us to distinguish users experiencing harm from those not experiencing harm based on their use patterns. Lastly, logistic binary analyses were conducted for each harm outcome, in order to quantify the risk for those use patterns identified in CART analyses to be predictive in classifying homogeneous user groups. These analyses were adjusted for age, gender, tobacco use, and other illegal drug use.

All statistical analyses were performed with SPSS statistical package version 20.0 and Microsoft Office Excel 2007.

### Ethics

The protocol was approved by the Ethics Committee of Hospital Clínic de Barcelona (HCB/2017/0795) according to the Helsinki Declaration (update Fortaleza 2013) and the national regulations.

## Results

### Descriptive Analyses

A total of 2,124 people who had used cannabis during the previous year answered the online survey and provided sufficient data regarding their cannabis use to be included in the analyses (see Figure 1); 68.6% were men, the mean age was 26.2 years old (SD 8.5), 58.1% were employed, and 51.6% had completed a university degree. Also, 75.6% reported at least one harm associated with chronic cannabis use in the literature. Type of cannabis used was often herbal (62.4%) and the route of administration was mainly smoked (96.3%). In our sample, 774 individuals (36.4% of the whole sample) reported risky alcohol use according to AUDIT-C (score >4); also, according to AUDIT-C, 63.7% of the sample reported use of alcohol at least twice a month. For more details about socio-demographic characteristics, patterns of cannabis use, and prevalence of other drug use, see Tables 1, 2.

**Table 1:** Socio-demographic and cannabis use characteristics of the sample.

**Categorical variables**		***N* (%)**
Gender		
•Males		1,457 (68.6)
•Females		652 (30.7)
•Other		15 (0.7)
Working status		
•Student		754 (35.8)
•Employment out of home		1,224 (58.1)
•Employment at home		32 (1.5)
•Unemployment		67 (3.2)
•Retired		22 (1.0)
•Leave		5 (0.2)
•Other		3 (0.1)
Studies	
•No studies		2 (0.1)
•Primary		34 (1.6)
•Secondary		969 (46.7)
•University		1071 (51.6)
Marital status		
•Single		13,232 (62.9)
•Couple		732 (34.8)
•Divorced		48 (2.3)
•Widow		2 (0.1)
Type of cannabis		
•Mainly Hash		311 (16.0)
•Mainly Herbal		1,241 (62.4)
•Both equally		407 (20.9)
•Other		14 (0.7)
RoA		
•Smoked		1,933 (96.3)
•Ingested		38 (1.9)
•Vaped		30 (1.5)
•Several routes or other routes		7 (0.3)
Purchase	
•Cannabis club		618 (30.0)
•Own production		227 (11.0)
•Dealer		554 (26.9)
•Friends		656 (31.8)
•Other/several ways		8 (0.4)
Cannabis use last 30 days		
•0 days		453 (21.4)
•1–19 days		783 (37.0)
•20 or + days		879 (41.6)
Years regular cannabis use		
•0–1		309 (18.6)
•2–10		923 (55.7)
•>10		425 (25.6)
Mg THC per day (last month)		
•0		462 (21.8)
•1–6		58 (2.7)
•7–14		975 (45.9)
•15–21		264 (12.4)
•>21		365 (17.2)
**Continuous variables**	**Mean**	**SD**
Age	26.2	8.5
Age onset first use (cannabis)	16.6	2.7
Age onset regular use (cannabis)	19.9	4.4
Years of regular use	7.7	7.9
Mg per day (according to Standard Joint Unit	13.6	14.1
Cognitive and behavioral problems	1.8	1.5

**Table 2:** Prevalence of other drug use in the sample.

**Drug use**	***N* (%)**
Tobacco use	847 (39.9)
Illegal drugs (lifetime)	1,142 (53.8)
Cocaine	
- Past	355 (16.7)
- Current	353 (16.6)
Opioids	
- Past	120 (5.6)
- Current	45 (2.1)
Amphetamine	
- Past	354 (16.7)
- Current	496 (23.4)
LSD	
- Past	302 (14.2)
- Current	162 (7.6)
Non-prescribed BZD	
- Past	147 (6.9)
- Current	124 (5.8)

### Univariate Analyses

Obtained data are described below, divided into the five independent variables: (1) milligrams THC per day of use during the last 30 days; (2) days of use during the last 30 days; (3) age of cannabis onset (first use); (4) age of regular cannabis onset; and (5) years of regular use. For more details, see Tables 3, 4.

**Table 3:** Patterns of cannabis use and harm correlations.

**Independent variables**	**SDS**	**AUDIT-C**	**PHQ-9**	**GAD-7**	**CAD-4**
Mg THC per day last month	0.394^*^	0.066^**^	−0.02	−0.038	0.011
Days of use last month	0.523^*^	0.070^***^	−0.42	−0.66	0.004
Age of onset use	−0.145^****^	−0.134^*^	0.024	0.044	−0.051
Age of onset regular use	−0.102^*****^	−0.121^*^	0.012	−0.015	−0.093
Years of cannabis use	0.082	−0.050^#^	−0.095^##^	−0.040	0.099^###^

*Pearson correlation test, only statistically significant (< 0.05):*

* *p < 0.001;*

** *p = 0.004;*

*** *p = 0.02;*

**** *p = 0.001;*

***** *p = 0.037;*

# *p = 0.049;*

## *p = 0.008;*

### *p = 0.038*.

**Table 4:** Cannabis use and harm categories univariate analyses.

		**Mg THC (means, SD)**	***t* (*p*)**	**Cohen's *d***	**Day of use last month (mean, SD)**	***t* (*p*)**	**Cohen's *d***	**Age onset (mean, SD)**	***t* (*p*)**	**Cohen's *d***	**Age onset regular use (mean, SD)**	***t* (*p*)**	**Cohen's *d***	**Years of Cannabis use (mean, SD)**	***t* (*p*)**	**Cohen's *d***
Sleep disorders	No	13.5 (13.9)	−1.029 (0.304)	N/A	13.7 (12.7)	1.380 (0.168)	N/A	16.5 (2.5)	−0.625 (0.532)	N/A	19.5 (4.2)	−0.094 (0.925)	N/A	7.6 (7.9)	0.687 (0.576)	N/A
	Yes	14.4 (15.4)			12.7 (12.9)			16.7 (3.7)		N/A	19.5 (5.5)		N/A	7.9 (8.4)		N/A
Mental health problems	No	13.5 (14.0)	−1.206 (0.234)	N/A	13.6 (12.7)	−0.429 (0.668)	N/A	16.6 (2.7)	0.626 (0.532)	N/A	19.5 (4.4)	<0.001	0.39	7.7 (7.9)	0.243 (0.808)	N/A
	Yes	16.8 (18.3)			14.4 (13.1)			16.3 (2.1)		N/A	18.2 (2.0)			7.3 (8.6)		N/A
Suicidal behavior	No	13.8 (14.1)	1.005 (0.315)	N/A	13.8 (12.7)	1.677 (0.094)	N/A	16.6 (2.5)	−0.863 (0.459)	N/A	19.5(4.4)	0.459 (0.687)	N/A	7.8 (7.9)	0.368 (0.226)	N/A
	Yes	13.0 (14.1)			12.6 (12.7)			16.7 (3.3)		N/A	19.3 (4.2)		N/A	7.2 (8.0)		N/A
Cognitive impairment	No	13.6 (14.1)	−0.410 (0.682)	N/A	13.4 (12.7)	−0.888 (0.374)	N/A	16.5 (2.5)	−0.442 (0.347)	N/A	19.6 (4.3)	1.134 (0.257)	N/A	7.9 (8.1)	2.070 (0.039)	0.12
	Yes	13.9 (14.1)			14.0 (12.8)			16.7 (3.3)		N/A	19.3 (4.6)		N/A	7.0 (7.5)		
Violence	No	13.6 (14.1)	−0.690 (0.490)	N/A	13.5 (12.7)	−1.047 (0.295)	N/A	16.6 (2.7)	0.375 (0.707)	N/A	19.5 (4.4)	0.171 (0.864)	N/A	7.6 (8.0)	−0.876 (0.381)	N/A
	Yes	14.8 (15.2)			15.1 (12.7)			16.4 (3.0)		N/A	19.4 (5.3)		N/A	8.5 (7.5)		N/A
Motor vehicle accidents	No	13.2 (13.9)	−3.514 (0.01)	0.26	13.1 (12.6)	−4.648 (<0.001)	0.33	16.7 (2.8)	5.948 (<0.001)	0.37	19.7 (4.6)	7.330 (<0.001)	0.44	7.3 (7.8)	−4.263 (<0.001)	0.33
	Yes	17.0 (15.7)			17.2 (12.6)			15.8 (2.0)			18.1 (2.7)			10.0 (8.3)		
Treatment for drug use	No	13.4 (13.8)	−2.247 (0.029)	0.36	13.5 (12.7)	−1.327 (0.185)	N/A	16.6 (2.7)	0.533 (0.596)	N/A	19.5 (4.3)	−0.269 (0.789)	N/A	7.4 (7.7)	−4.064 (<0.001)	0.66
	Yes	19.8 (20.4)			15.9 (13.6)			16.3 (3.5)			19.8 (7.1)			13.2 (10.0)		

*t Student independent samples*.

Violence, gambling, sleep disorders, cognitive impairment, suicidal impulses, anxiety, and depression were not associated with any independent variable.

#### Acute Exposure Measured by Milligrams Delta-9-THC per Day of Use During the Last Month

Mg of THC correlated with SDS score (*r* = 0.394; *p* < 0.001) and AUDIT-C score (*r* = 0.066; *p* = 0.004). The average daily intake of THC in the past 30 days was higher for those users who experienced a motor vehicle accident after cannabis use (<6 h) (13.2 vs. 17.0, *p* = 0.01; Cohen's *d* = 0.26) or received treatment for substance use disorder (SUD) (13.4 vs. 19.8, *p* = 0.029, Cohen's *d* = 0.36).

#### Acute Exposure Measured by Days of Cannabis Use During the Previous 30 Days

DU correlated with SDS score (*r* = 0.523; *p* < 0.001) and AUDIT-C score (*r* = 0.070; *p* < 0.02). Mean of days of use was higher for those users who experienced a motor vehicle accident after cannabis use (<6 h) (13.1 vs. 17.2, *p* < 0.001, Cohen's *d* = 0.33).

#### Early Exposure Measured by Age of Onset (First Use of Cannabis)

AOf correlated inversely with SDS score (*r* = −0.145, *p* = 0.001) and AUDIT-C score (*r* = −0.134; *p* < 0.001). The mean age of onset was lower for those users who had motor vehicle accidents (15.8 vs. 16.7, *p* < 0.001, Cohen's *d* = 0.37).

#### Early Exposure Measured by Age of Onset (Regular Cannabis Use)

AOr correlated inversely with SDS score (*r* = −0.102; *p* = 0.037) and AUDIT-C score (*r* = −0.121; *p* < 0.001). The mean age of onset of regular use was lower for those users who experienced motor vehicle accidents (19.7 vs. 18.1, *p* < 0.001, Cohen's *d* = 0.44). It was also lower for those users who reported history of mental health disorders (19.5 vs. 18.2, *p* < 0.001, Cohen's *d* = 0.39).

#### Chronic Exposure Measured by Years of Regular Use

YRCU correlated with CAD (*r* = 0.099; *p* = 0.038) and inversely with PHQ-9 (*r* = −0.095, *p* = 0.008) and AUDIT-C (*r* = −0.050, *p* = 0.049). The mean of years of regular use was higher for those users who had cognitive impairment (7.9 vs. 7.0, *p* = 0.039, Cohen's *d* = 0.12), experienced a motor vehicle accident after cannabis use (<6 h) (10.0 vs. 7.3, *p* < 0.001, Cohen's *d* = 0.33), or received treatment for SUD (13.2 vs. 7.4, *p* < 0.001, Cohen's *d* = 0.66).

**Figure 2:**
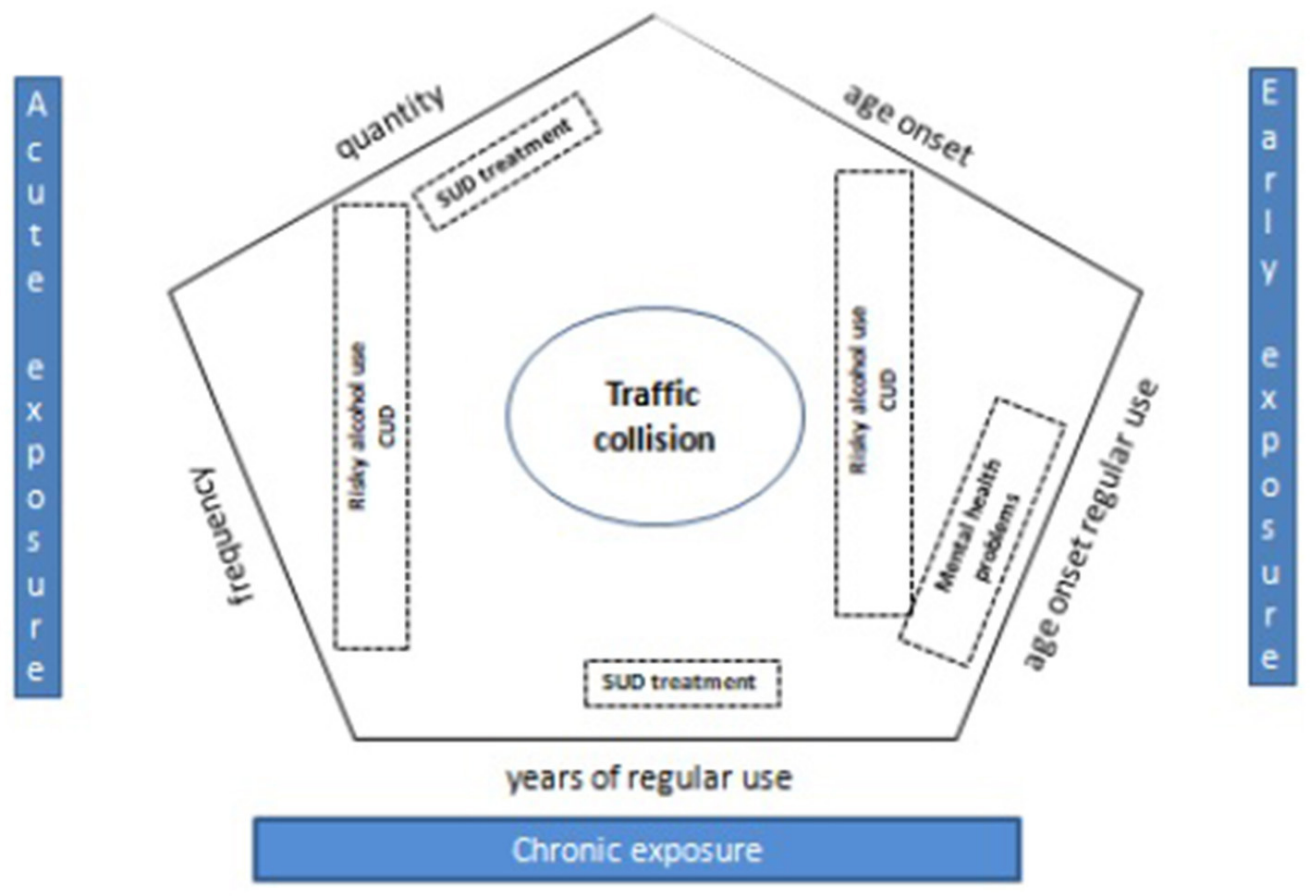
Summary of results: indicators of acute, early and chronic cannabis exposure and associations with health harms.

Figure 2 provides a summary of results.

### CART Analyses and Logistic Binary Regression Analyses

#### Harm 1: SDS Score > 4

In the CART analysis, we included those variables associated with SDS score > 4 in the univariate analysis (frequency of use, quantity of use, age of first use, and age of onset regular use).

**Figure 3:**
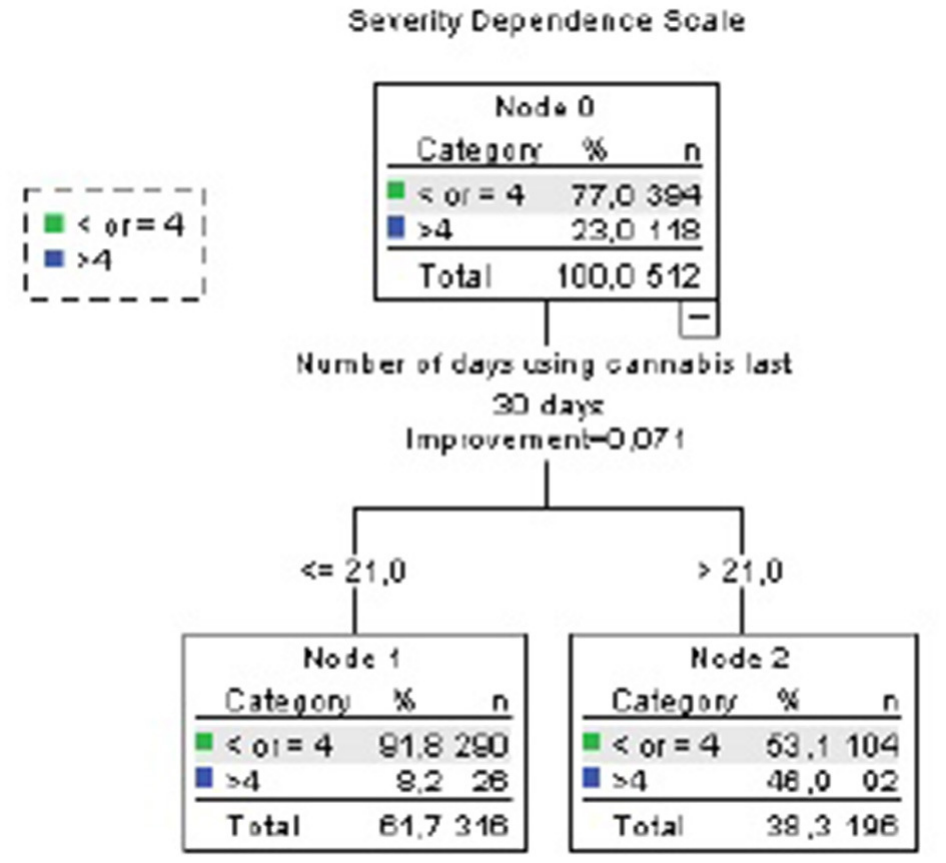
CART analysis (Severity Dependence Scale). Accuracy 77%. Independent variables included: mg THC per day of use last month, days of use last month, age of first use, age of onset of regular use.

According to the CART analysis, among survey respondents using cannabis >21 days in the last month, 46.9% had an SDS score > 4 (node 2, Figure 3), as compared to only 8.9% of those who used cannabis less frequently (node 1, Figure 3; whole sample: 23%). Other variables (quantity, age of first use, and age of onset regular use) did not explain additional variance.

After adjusting for age, gender, use of other illegal drugs, and tobacco use in a Logistic Binary Regression Analysis, those who used cannabis >21 days per month had eight times higher probability of SDS >4 (OR 8.33, 95% CI 4.91–14.16, *p* < 0.001).

#### Harm 2: AUDIT-C Score > 4, Suggestive of Risky Alcohol Use

In the CART analysis, we included those variables associated with AUDIT C > 4 (which is suggestive of risky alcohol use) in the univariate analysis (frequency of use, quantity of use, age of first use, and age of onset regular use).

**Figure 4:**
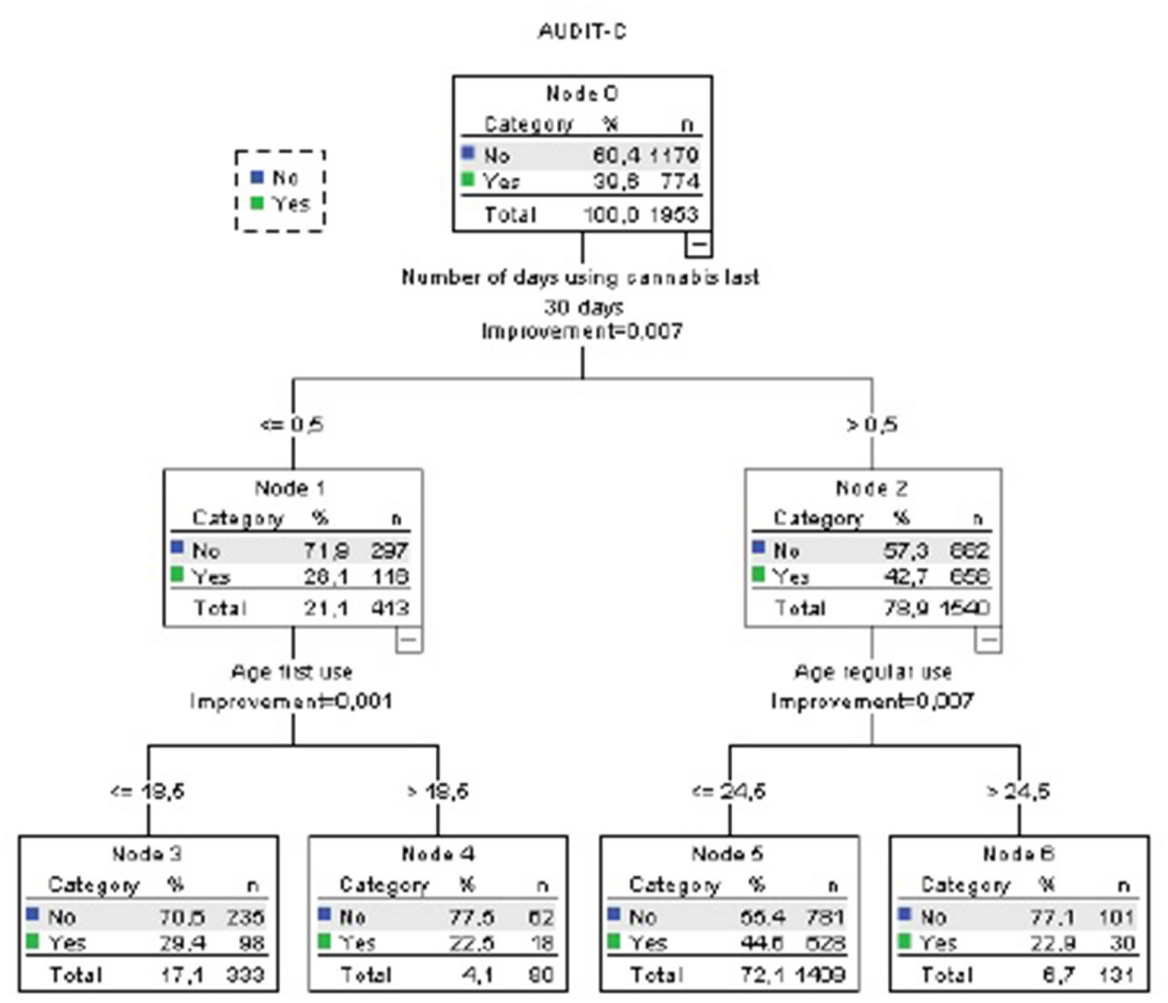
CART analysis (Alcohol Use Disorder Identification Test-C). Accuracy 60.4%. Independent variables included: mg THC per day of use last month, days of use last month, age of first use, age of onset of regular use.

According to the CART analysis, among survey respondents using cannabis at least 1 day in the last month and started using cannabis regularly before 25 years old, 44.6% had AUDIT-C positive (node 5, Figure 4), as compared with 39.6% of whole sample. Age of first use > 18.5 years old was associated with a lower risk of AUDIT-C > 4 in those who did not use cannabis in the last month (22.4%). Quantity of use did not explain additional variance.

After adjusting for age, gender, use of other illegal drugs, and tobacco use in Logistic Binary regression analyses, those who used cannabis at least once the last month had 1.5 times higher probability of AUDIT-C positive (OR 1.56, 95% CI 1.21–2.01, *p* = 0.01) while age of onset of regular use was not statistically significant in this model. However, a combination of both use indicators (regular use <25 years and using cannabis at least once per month) was associated with increased risk of being AUDIT-C positive, as compared with those who had not any of them (OR 1.33, 95% CI 1.12–1.57, *p* = 0.001).

#### Harm 3: Motor Vehicle Accident <6 h After Using Cannabis

In the CART analysis, we included those variables associated with motor vehicle accidents in the univariate analysis (frequency of use, quantity of use, age of first use, age of onset regular use, and year of regular use).

**Figure 5:**
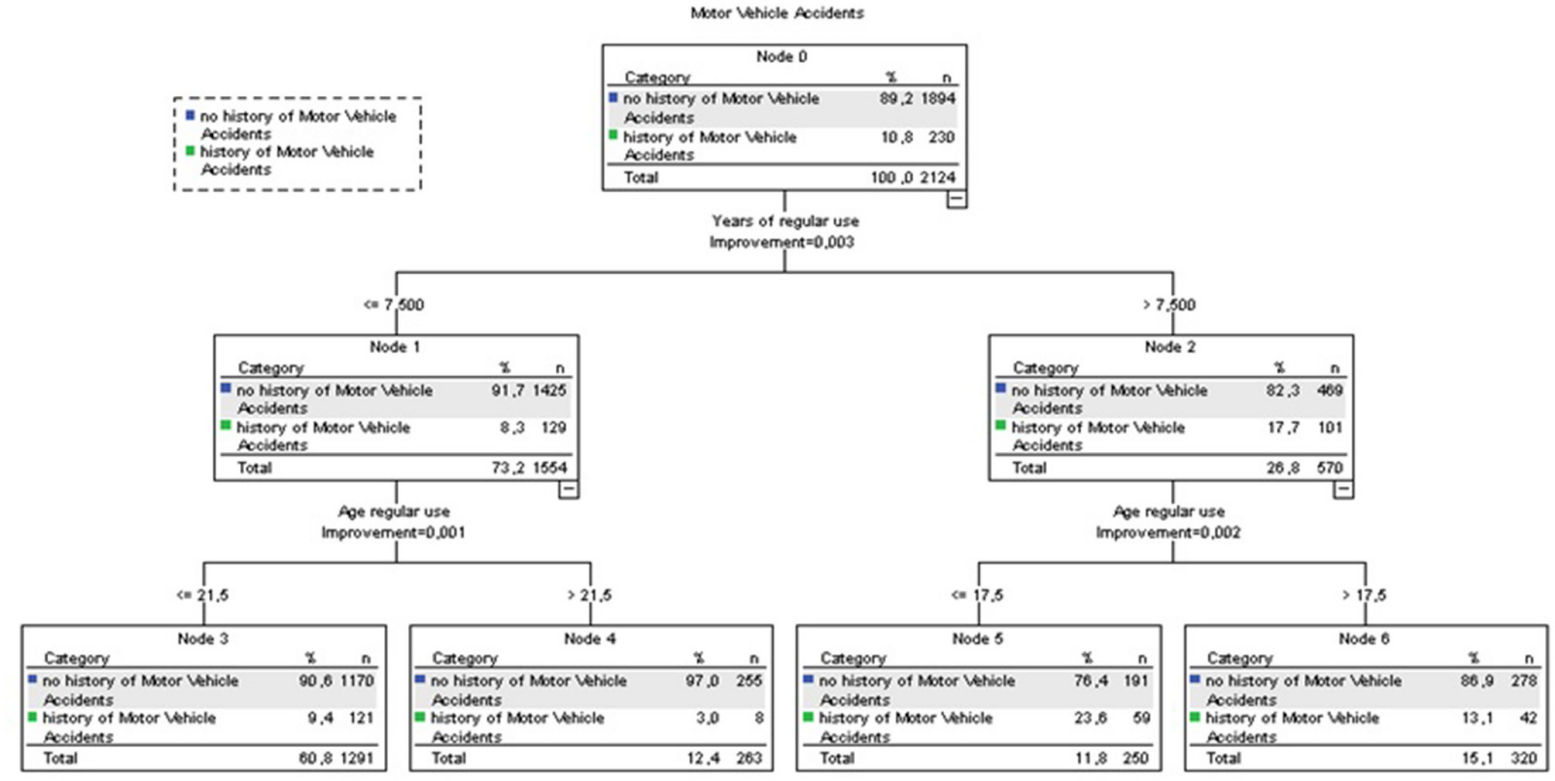
CART analysis (Motor Vehicle Accidents). Accuracy 89.2%. Independent variables included: mg THC per day of use last month, days of use last month, age of first use, age of onset of regular use, years of regular use.

According to the CART analysis, among survey respondents using cannabis for at least 7.5 years and initiated regular cannabis use before 18 years old, 23.6% had motor vehicle accidents (node 5, Figure 5), as compared with 3% of those who used cannabis <7.5 years and started cannabis use after 21.5 years old (node 4, Figure 5; whole sample: 10.8%). Other variables (quantity, frequency of use, and age of onset) did not explain additional variance.

After adjusting for age, gender, use of other illegal drugs and tobacco use in Logistic Binary regression analyses, those who use cannabis for at least 7.5 years had 1.4 times higher probability of motor vehicle accidents (OR 1.4, 95% CI 1.04–1.96, *p* = 0.030) and those who use cannabis regularly before 18 years old had 1.9 times higher probability of motor vehicle accidents (OR 1.93, 95% CI 1.43–2.60, *p* < 0001). Combination of both use indicators (regular use <18 years and using cannabis at least during 7.5 years) was associated with higher probability of motor vehicle accidents (OR 1.81, 95% CI 1.41–2.32, *p* < 0.0001) compared with those who did not report these use patterns.

#### Harm 4: History of Treatment for SUDs

In the CART analysis, we included those variables associated with history of treatment for SUDs in the univariate analysis (frequency of use and year of regular use).

**Figure 6:**
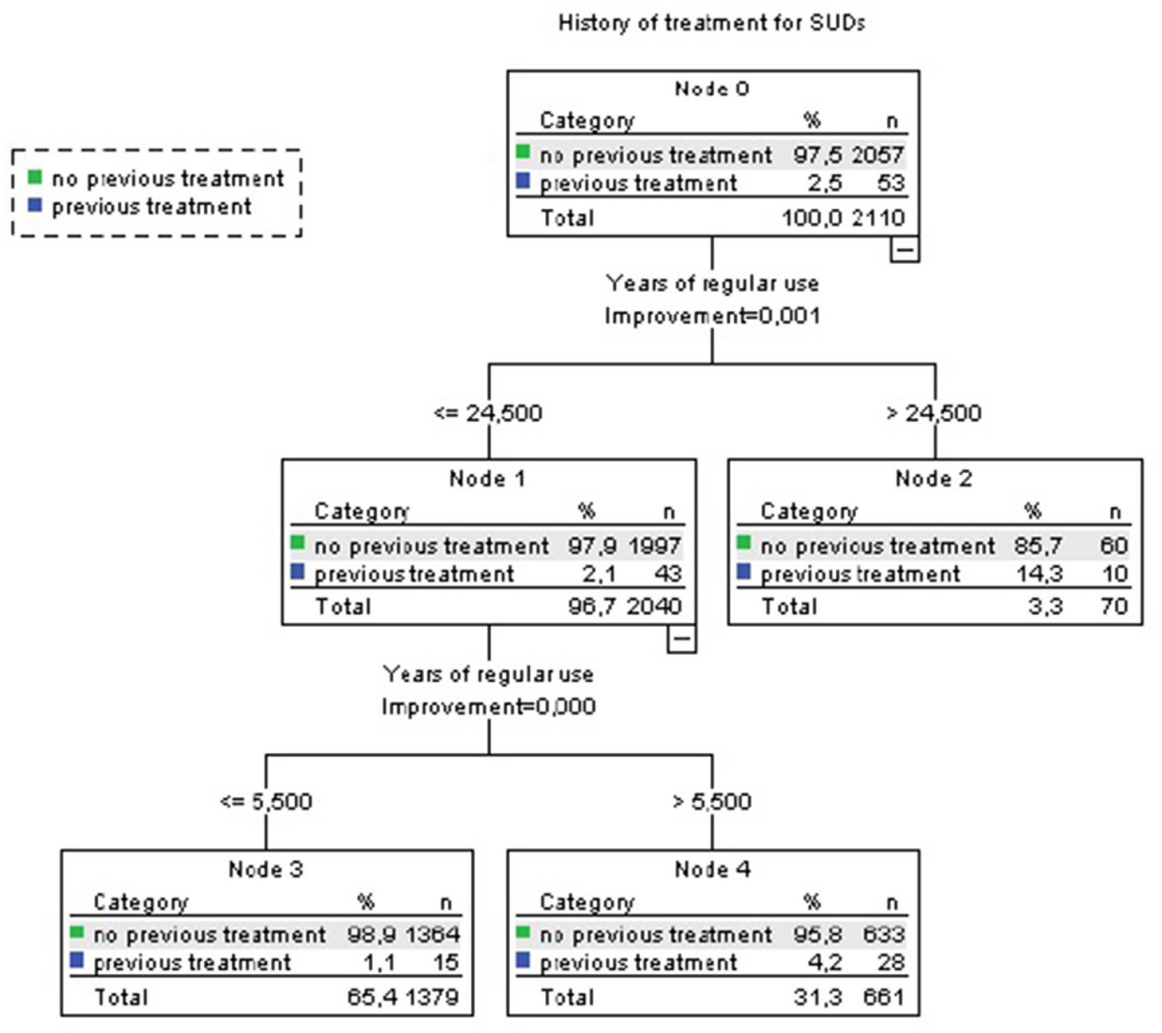
CART analysis (Treatment for SUDs). Accuracy 97.5%. Independent variables included: mg THC per day of use last month, years of regular use.

According to the CART analysis, among survey respondents using cannabis during at least 24.5 years, 14.3% had received treatment for SUDs (node 2, Figure 6), as compared with 1.1% of those who used cannabis during <6 years (node 3, Figure 6; whole sample: 2.5%). Quantity of use did not explain additional variance.

After adjusting for age, gender, use of other illegal drugs, and tobacco use in Logistic Binary regression analysis, years of regular use was not associated with treatment for SUDs.

#### Sensitivity Analyses (Those Who Reported Regular Use Since Before 18 Years Old; n = 517)

**Figure 7:**
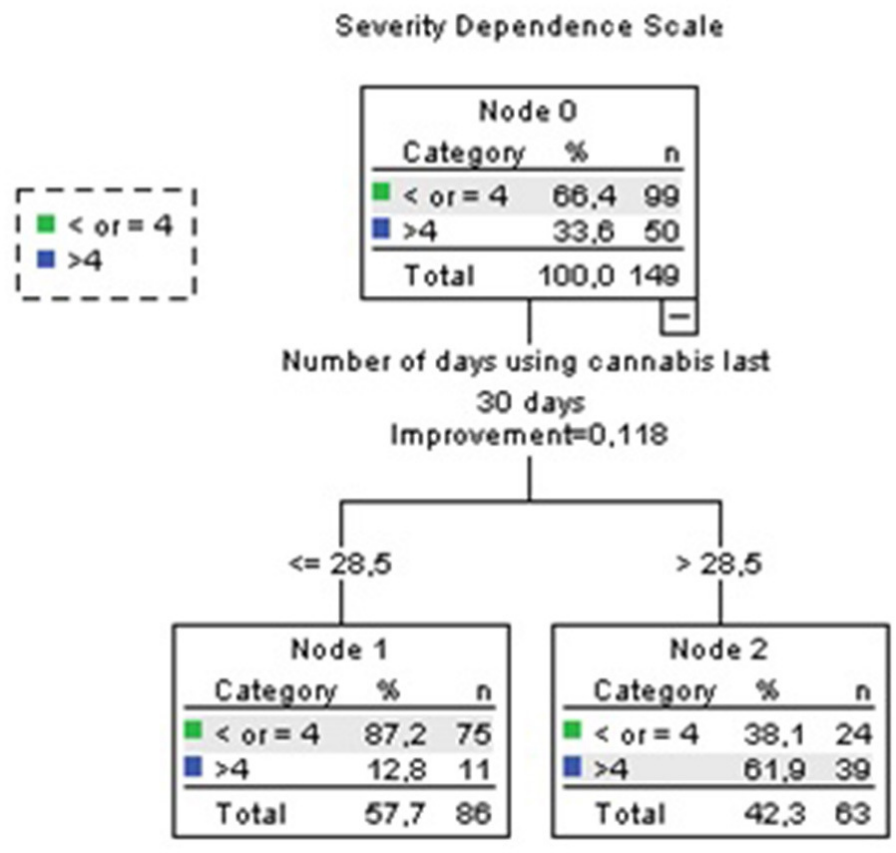
Sensitivity analyses. Accuracy 76.5%.

In these analyses, we did not include variables of age of onset of regular use because it was the selection criteria for this subsample. SDS score correlated with mg THC per day of use last month (0.365, *p* < 0.001) and days of use last month (0.474, *p* < 0.001). CART analyses identified a subgroup of higher risk of SDS >4 (which is indicative of a cannabis use disorder) in those who used cannabis at least 28.5 days per month (61.9 vs. 33.6% of whole subsample, see Figure 7). PHQ score correlated inversely with years of regular use (−0.148, *p* = 0.018). Previous treatment for SUD was associated with years of regular use (mean 9.7, SD 7.8 no previous treatment vs. mean 15 years, SD 9.3 previous treatment, *p* = 0.002). History of motor vehicle accidents was associated with years of regular use (mean 9.4, SD 7.8 no motor vehicle accidents vs. mean 12.9, SD 8.2 motor vehicle accidents, *p* < 0.001). Cognitive impairment was associated with years of regular use. In this case, longer use was associated with lower probability of cognitive impairment (mean 10.6, SD 8.3 no cognitive impairment vs. mean 8.4, SD 6.8 cognitive impairment; *p* = 0.02). Other outcomes were not associated with any patterns of cannabis use.

## Discussion

The relationship between cannabis use and health harms is a complex phenomenon. Among all these potential health harms, cognitive, and behavioral consequences are the best studied and described in the literature. This study analyzes the relationship between acute, early, and chronic exposure and several indicators of cognitive and behavioral harm. The vast majority of previous research did not include several dimensions of pattern of use; specifically, they did not take into account quantity and the combination of early, acute, and chronic exposure. Clarifying the influence of each of these dimensions on cognitive and behavioral harm is a necessary first step to eventually develop instruments that might allow clinicians to straightforwardly identify those users who are more prone to suffer from psychological or behavioral harm related to cannabis and thus providing targeted further assessment and treatment.

Our results suggest that using cannabis 3 out of 4 days increases eight times the probability of scoring 4+ on the SDS, which is indicative of a cannabis use disorder. Also, in our sample, a start of regular cannabis use before 25 years old combined with using cannabis at least once per month is associated with higher probability of risky alcohol use. Besides, a start of regular cannabis use before 18 years old combined with a period of regular use of at least 7.5 years was associated with 80% higher odds of motor vehicle accidents. On the other hand, results were ambiguous regarding the role that age of first use and milligrams of THC per day of use might play regarding cannabis-related harms.

In our sample, we could not find a clear association between acute, chronic, or early cannabis exposure and violence, gambling, sleep disorders, cognitive impairment, suicidal impulses, anxiety, and depression. Several arguments rise to potentially explain these results. First, violence and suicidal impulses were evaluated through *ad hoc* questions because otherwise the questionnaire would have been too long. Unvalidated questions may not detect existing harms in these dimensions. Second, the cannabis use indicators included in this study might not be sufficient to explain these harms. Other variables [e.g., number of heavy cannabis use days and high-potency cannabis (8)] might explain better those harm indicators. Third, literature shows a different level of evidence between cannabis use and harms. While the relationship between motor vehicle accidents or CUD and cannabis use is well-established, the relationship between gambling, violence, and sleep disorders is less clear (7). Moreover, the mean age of the sample is relatively low (26.2 years old), while some harms (such as suffering a motor vehicle accident or receiving treatment for substance use) might need longer chronic cannabis exposure (35, 36).

Our findings that frequency of use is associated with severity of dependence are consistent with previous research. For instance, a recent systematic review concluded that the most consistent predictive factors of cannabis dependence were an early onset of cannabis use, frequent use, and prior drug involvement. Comorbid mental disorders like affective disorders, anxiety disorders, and alcohol-related disorders also seem to predict first incidence of cannabis dependence (18). Our results suggest that frequency of cannabis use of 3 out of every 4 days (or more than 21 days per month) increased severity of cannabis dependence (SDS >4). This would be a higher threshold than the ones proposed in most previous literature. For instance, a recent cross-sectional study pointed to a threshold of cannabis use at least two to three times a month for increased probability of suffering a cannabis use disorder or psychosocial functioning cannabis-related problems when quantity of use was at least one joint (37). Also, a cohort study of Australian secondary students suggested a threshold of weekly cannabis use during adolescence for increased probability of cannabis use disorders (38). Nonetheless, other previous studies had already pointed to near-daily or daily frequency of cannabis use for increased risk of cannabis use disorders (39, 40). According to previous studies, younger age at first cannabis use seems to be of crucial importance to the development of dependence. Previous studies stated that an early onset of cannabis use and persistent cannabis use were markers of increased risk of cannabis dependence. Adolescents with daily and weekly use in adolescence (aged 14–17) were more prone to develop dependence later at 24 years of age (41).

Our findings also suggest that a start of regular cannabis use before the age of 25 combined with using cannabis at least once per month might predict higher probability of risky alcohol use. The relationship between alcohol use and increased risk of cannabis-related health harms has been extensively described in previous literature. For instance, in one study, cannabis use appeared to be a marker of cannabis dependence symptoms only in participants who consumed alcohol frequently or in large amounts (42). In another study, lifetime diagnosis of alcohol dependence has been found to increase the risk for lifetime cannabis dependence (43). On the other hand, although alcohol and marijuana/cannabis are frequently used simultaneously, studies suggest that acute negative consequences of co-use are associated with using more than one alcohol product (44).

Particularly, the fact that our results suggest that an early onset of regular cannabis use and frequent use are associated with increased probability of risky alcohol use are in line with a recent latent trajectory analysis study of a longitudinal birth cohort that suggested that individuals with early onset of cannabis use and at least weekly use by age 20 had increased odds of suffering from alcohol dependence (45). Conversely, another cross-sectional study proposed that individuals that consumed cannabis more than once per week in the last 30 days had a higher probability of risky alcohol use (46).

Our data tentatively imply that cannabis users who experience mental health problems might tend to initiate regular cannabis use at earlier ages, which is consistent with previous studies on the relationship between early cannabis exposure and psychiatric disorders, especially psychosis (20).

Also, in our sample, although the respondents who had used cannabis for 24.5 years had received treatment for SUDs more frequently than those who only used cannabis for periods of <6 years, after adjusting for age, gender, use of other illegal drugs, and tobacco use, years of regular use was not associated with treatment for SUDs. However, several previous studies imply that cannabis use is associated with use of other substances, both concurrent (46) and in later stages of life (47). Also, previous literature described a doubling in the number of individuals entering specialized drug treatment for cannabis-related problems for the first time in EU between 2003 and 2014 (48). Similar increases have been less consistent for other illicit drugs, and cannabis problems appear to be responsible for an increasing percentage of all new drug treatment demands (49). Considering all that, it would be coherent to assume that individuals that consume higher THC doses or that have been consuming cannabis for longer periods of time are more prone to be treated for any SUD.

Sensitivity analyses of those who reported regular use since before 18 years old (*n* = 517) showed a high risk of SDS >4 (which is indicative of a cannabis use disorder) among those who smoked daily or almost daily (62% of them). As in the whole sample, interpretation of the association between patterns of use with cognitive impairment and PHQ score is challenging. In this subsample, motor vehicle accidents and previous treatment were only associated with years of regular use, with little impact of quantity or frequency. This might translate into years of regular use having a specific weight in the impact on motor vehicle accidents and previous treatment among those who started to use cannabis regularly earlier being quantity and/or frequency more relevant for those who started later.

Lastly, we should note that according to our data, a start of regular cannabis use before 18 years old combined with a period of regular use of at least 7.5 years is associated with higher probability of motor vehicle accidents. A systematic review published in 2010 linked fatal motor vehicle accidents mostly with frequency and quantity of use criteria, specifically more than 50 occasions of use by age 18 or smoking more than 10 joints per week (25). More so, the association between cannabis use and motor vehicle accidents has been described extensively in the literature (7). Previous studies reported that marijuana use by drivers is associated with a significantly increased crash risk. The crash risk appears to increase progressively with the dose and frequency of marijuana use (50). Most past studies highlighted the relationship between acute cannabis use and the collision. For instance, in a sample of 860 injured drivers presenting to Canadian emergency departments due to a traffic collision, controlling for other substance use and acute cannabis consumption, measured through blood sample or self-report, was associated with a 4-fold increase in the risk of a traffic collision, and the association remained when employing a usual frequency control condition (51). Interestingly, our results suggest that not only acute cannabis exposure but also early and chronic exposure could point to higher risk for crash accidents.

## Limitations and Strengths

Several limitations of our study are pointed out. On the one hand, this is a cross-sectional study, with well-known limitations to assess causality between patterns of cannabis use and health-related harms. Also, although collection of data with a self-administered questionnaire accessible through an online survey allows the recruitment of a big sample size, the sample may be biased by self-selection, i.e., persons with problem of cannabis use may be differentially prone to participate, requiring further validation of results (52). Our results were obtained only from people living in Spain (Europe), which might hamper generalization of the results to other sociocultural contexts. In addition, the pattern of cannabis use and also other substance use could only be assessed by self-reported measures. Nonetheless, literature shows that self-reported substance use, including cannabis, correlates in a fairly precise manner to positive urine toxicology tests (53). In fact, some authors have stressed that history and scales are more reliable than drug screening for cannabis use detection (54, 55). Furthermore, the Food and Drug Administration (FDA) and European Medicines Agency (EMA) recognize self-reporting as a tool for drug approval during clinical trials. Besides, to our knowledge, this is the first survey that assessed cannabis use and health-related harms that applied a cannabis dose standardized measure [the Standard Joint Unit (27)] to convert individual self-reports of cannabis use (joints and grams used the last month and money spent on cannabis during the last month) to milligrams of cannabis main psychoactive constituent (THC).

Also, experiencing some of the behavioral harms assessed (such as motor vehicle accidents or receiving treatment for substance use) is highly age-dependent, so results regarding the relationship of these harms with dimensions of pattern of use that are also age-dependent, such as years of chronic use, should be interpreted with caution.

To summarize, the cross-sectional design of our study does not allow us to establish causality or to assuredly define the threshold for frequency, quantity, age of first use, age of initiation of regular use, or years of regular use that affect harm, but our results increase the evidence in favor of considering not just frequency of use but also other dimensions of cannabis use in both research and clinical practice.

The relationship between pattern of cannabis use and neuropsychological harm is a complex phenomenon, even more so when considering that cannabis psychoactive constituents disrupt the natural functioning of the endocannabinoid system, that affects both central nervous system (CNS) and peripheral processes and plays a role on anxiety, depression, neurogenesis, reward, cognition, learning, and memory (56, 57). Further, research efforts with longitudinal data should be made in order to have a better understanding of how all these five dimensions interact together to determine neuropsychological harms.

## Conclusions

Using cannabis 3 out of 4 days might increase up to eight times the probability of scoring 4+ on the Severity Dependence Scale, which is indicative of a cannabis use disorder. Also, a start of regular cannabis use before 25 years old combined with using cannabis at least once per month might increase probability of risky alcohol use. Besides, a start of regular cannabis use before 18 years old combined with a period of regular use of at least 7.5 years was associated with increased risk probability of motor vehicle accidents. The pattern of cannabis use should be carefully and widely evaluated—not just the frequency of use but also other dimensions—in research to assess cannabis-related harms. In order to have a better understanding of what kind of cannabis use predicts higher risk for experiencing cognitive and behavioral harms, future research with longitudinal data needs to determine the single independent contribution of each cannabis use indicator to experience harm. This could allow determining cutoffs for the relevant indicators, since it offers healthcare providers a practical tool to identify consumers at risk. This would also facilitate early preventive strategies and better monitoring of treatment interventions aimed at risk and harm reduction.

## Data Availability Statement

The datasets presented in this article are not readily available because database is under analysis for other studies. Requests to access the datasets should be directed to Eugénia Campeny, campeny@clinic.cat.

## Ethics Statement

The studies involving human participants were reviewed and approved by the Ethics Committee of Hospital Clínic de Barcelona (HCB/2017/0795) according to the Helsinki Declaration (update Fortaleza 2013) and the national regulations.

## Author Contributions

MB-O, AG, EC, and HL-P designed the study. EC, HL-P, CO, and MB-O wrote the first draft of the manuscript. All the other authors reviewed and approved the final paper.

## Conflict of Interest

The authors declare that the research was conducted in the absence of any commercial or financial relationships that could be construed as a potential conflict of interest.

## Publisher's Note

All claims expressed in this article are solely those of the authors and do not necessarily represent those of their affiliated organizations, or those of the publisher, the editors and the reviewers. Any product that may be evaluated in this article, or claim that may be made by its manufacturer, is not guaranteed or endorsed by the publisher.

## Abbreviations

AOf: Age of onset (first use of cannabis)
AOr: Age of onset (regular cannabis use)
CUD: Cannabis use disorder
DU: Days of cannabis use during the previous 30 days
YRCU: Years of regular use.
