# Supplementary material for: Early, Chronic, and Acute Cannabis Exposure and Their Relationship With Cognitive and Behavioral Harms
Source: Front Psychiatry. 2021 Aug 9;12:643556. doi: 10.3389/fpsyt.2021.643556 (PMC8381725; doi:10.3389/fpsyt.2021.643556)
Supplement: Supplementary file 1 [file Data_Sheet_1.PDF]

Edad: ☐

Género:

- Masculino
- Femenino
- Otro

Lugar de residencia:

- España
- Fuera de España

Nivel de estudios:

- **Sin estudios** o estudios **primarios incompletos** (menos de 6 años o cursos de primaria aprobados)
- Estudios **primarios completos** (6º EGB aprobado, certificado de escolaridad, 6 años o cursos de primaria aprobados)
- Estudios **secundarios 1ª etapa** (8º EGB aprobado, graduado escolar, bachillerato elemental, formación profesional de 1º grado o ciclos formativos de grado medio aprobados, ESO aprobada, 4 años o cursos de secundaria aprobados)
- Estudios **secundarios 2ª etapa** (BUP, bachillerato superior, bachillerato LOGSE, bachillerato LOMCE, COU, PREU, formación profesional 2º grado o ciclos formativos de grado superior, 5 o más años o cursos de secundaria aprobados)
- Estudios **universitarios medios** (diplomatura, magisterio, arquitectura o ingeniería técnica, grado)
- Estudios **universitarios superiores** (licenciatura, arquitectura o ingeniería superior, doctorado, master)
- Ns/Nc

¿Cuál es su situación laboral?

- Trabaja sólo en las tareas de casa
- Trabaja (sin incluir tareas de casa)
- Está en paro
- Es pensionista o está jubilado

Estado civil:

- Soltero/a
- Casado/a
- Separado/a o divorciado/a
- Viudo/a

¿Consume tabaco de forma habitual?

- Tabaco:
    - Sí
        - Cantidad de cigarrillos al día:

        - Edad de inicio del consumo regular:
    - No
- Alcohol:
    - ¿Con qué frecuencia consume alguna bebida alcohólica?
        - Nunca
        - Una o menos de una vez al mes
        - 2 a 4 veces al mes
        - 2 o 3 veces a la semana
        - 4 o más veces a la semana
    - ¿Cuántas consumiciones de bebidas alcohólicas suele realizar en un día de consumo normal?
        - 1 o 2
        - 3 o 4
        - 5 o 6
        - 7 a 9
        - 10 o más
    - ¿Con qué frecuencia toma 6 o más bebidas alcohólicas en un solo día?
        - Nunca
        - Menos de una vez al mes
        - Mensualmente
        - Semanalmente
        - A diario o casi a diario
- ¿Alguna vez ha consumido drogas ilegales?
    - Si
    - No
- ¿Consume alguna de las siguientes substancias ilegales?

**Sustancia**	**He consumido o he tenido consumos puntuales**	**Consumo o tengo consumos puntuales**
Cocaína		
Opioides		
Anfetaminas		
LSD		
Sedantes sin prescripción médica (lexatin, trankimazín, ofidal, valium, tranxilium, stilnox, noctamid)		

- ¿En cuántas ocasiones en los últimos 12 meses?

A continuación le vamos a hacer una serie de preguntas referentes a su consumo de cannabis.

- ¿Cuántos días ha consumido cannabis en **el último mes**?
    - Especificar:
- En un día de consumo…
    - ¿Cuántos euros se gasta?
        - 1 €
        - 2 €
        - 3 €
        - 4 €
        - Más, especificar:
    - ¿Cuántos gramos consume?
        - 0,25 gr
        - 0,50 gr
        - 0,75 gr
        - 1 gr
        - Más, especificar:
    - ¿Cuántos porros fuma?
        - 1 porro
        - 2 porros
        - 3 porros
        - 4 porros
        - Más, especificar:
- ¿Qué tipo de cannabis consume habitualmente?
    - Hachís (chocolate, piedra…)
    - Marihuana (maría, hierba)
    - Otros. Especificar:
- Cuando se lía el porro, ¿qué porcentaje del mismo es tabaco?

□ 0% □ 25% □ 50% □ 75% □ 100%

- ¿Cuál es su forma preferente para obtener cannabis?
    - Asociación de usuarios de cannabis
    - Cultivo propio
    - Vendedor (camello)
    - Otros. Especificar:
- ¿Cómo suele consumir el cannabis?
    - Fumado
    - Ingerido
    - Otros. Especificar:
- ¿A qué edad consumió su primer porro?
    - Especificar:

- ¿A qué edad empezó a consumir regularmente?
  - Especificar:
- **¿Ha recibido o está en tratamiento por alguna de las siguientes substancias?**

	**Sí**	**No**
**Alcohol**		
**Cocaína**		
**Cannabis**		
**Heroína**		
**Otras:**		
**No**		

A continuación le vamos a preguntar acerca del estado de su salud mental.

1.

**Durante las últimas 2 semanas, ¿qué tan seguido ha tenido molestias debido a los siguientes problemas?**	**Ningún día**	**Varios días**	**Más de la mitad de los días**	**Casi todos los días**
1. Poco interés o placer en hacer cosas	0	1	2	3
2. Se ha sentido decaído(a), deprimido(a) o sin esperanzas	0	1	2	3

Trastornos Depresivos (en caso de que la conteste afirmativo (1, 2 o3) en las dos preguntas de la tabla anterior) – PHQ-9. Preguntar entero:

Señale con qué frecuencia, en los **últimos 15 días**, se ha sentido molesto debido a los siguientes problemas.	**Ningún día**	**Varios días**	**Más de la mitad de los días**	**Casi todos los días**
Poco interés o placer en hacer cosas	0	1	2	3
Se ha sentido triste, deprimido/a o sin esperanzas	0	1	2	3
Ha tenido dificultad para quedarse o permanecer dormido/a o ha dormido demasiado.	0	1	2	3
Se ha sentido cansado/a o con poca energía	0	1	2	3

Sin apetito o ha comido en exceso	0	1	2	3
Se ha sentido mal con usted mismo/a, o ha sentido que es un fracaso o que ha quedado mal con usted mismo/a o con su familia.	0	1	2	3
Ha sentido dificultad para concentrarse en ciertas actividades, como leer el periódico o mirar la televisión	0	1	2	3
Se ha movido o hablado tan lento que otros podrían haberlo notado, o por el contrario, ha estado tan inquieto/a o agitado/a que ha estado moviéndose mucho más de lo normal.	0	1	2	3
Ha pensado que estaría mejor muerto/a, o ha tenido pensamientos de herirse a sí mismo/a de algún modo.	0	1	2	3

2.

**Durante las últimas 2 semanas, ¿qué tan seguido ha tenido molestias debido a los siguientes problemas?**	**Ningún día**	**Varios días**	**Más de la mitad de los días**	**Casi todos los días**
1. Se ha sentido nervioso(a), ansioso(a) o muy alterado(a)	0	1	2	3
2. No ha podido dejar de preocuparse o controlar la preocupación	0	1	2	3

<u>Trastornos de ansiedad</u> (en caso de que la conteste afirmativo (1, 2 o3) en las dos preguntas de la tabla anterior) – GAD-7. Preguntar entero:

**Señale con qué frecuencia ha sufrido los siguientes problemas en los últimos 15 días.**	**Nunca**	**Menos de la mitad de los días**	**Más de la mitad de los días**	**Casi todos los días**
Se ha sentido nervioso/a, ansioso/a o muy alterado/a	0	1	2	3
No ha podido dejar de preocuparse o controlar la preocupación	0	1	2	3
Se ha preocupado excesivamente por diferentes cosas	0	1	2	3
Ha tenido dificultad para relajarse	0	1	2	3

Se ha sentido tan intranquilo que no podía estar quieto/a	0	1	2	3
Se ha irritado o enfadado con facilidad	0	1	2	3
Ha sentido miedo o preocupación, como si fuese a suceder algo terrible.	0	1	2	3

3. **¿Alguna vez en la vida le han diagnosticado alguna de las siguientes enfermedades? Puede seleccionar más de una opción de respuesta.**

**Trastorno depresivo** (Depresión mayor, distimia…)
**Trastorno de ansiedad** (t. ansiedad generalizada, t de pánico, agorafobia, fobia específica, fobia social, t. estrés postraumático…)
**Trastorno psicótico** (esquizofrenia, t. psicótico breve, trastorno delirante, t. esquizoafectivo…)
**Trastorno bipolar** (episodio maníaco, ep. hipomaníaco, t. bipolar tipo I, tipo II, ciclotimia…)
**Otros: especificar:**
**No**

4. **En el último año, ¿Ha jugado a juegos de azar o ha participado en apuestas? (apuestas deportivas o de otro tipo, máquinas tragaperras, juegos online…)**
    a. Sí
    b. No

<u>Juego patológico</u> (en caso de afirmativo en pregunta 3) – CAD-4. Preguntar todas las 4 cuestiones siguientes:

1. **Ha tenido usted la sensación de que debería reducir su conducta de juego?**
    a. Sí
    b. No
2. **¿Niega u oculta su verdadera conducta de juego ante las posibles críticas de los demás sobre sus supuestos excesos?**
    a. Sí
    b. No
3. **¿Ha tenido usted problemas psicológicos, familiares, económicos o laborales a causa del juego?**
    a. Sí
    b. No
4. **¿Se siente con frecuencia impulsado irremediablemente a jugar a pesar de sus problemas?**

a. Sí
b. No

5. En los últimos 12 meses, ¿con qué frecuencia le ha ocurrido algo de lo que se describe a continuación?

**Durante las últimas 2 semanas, ¿qué tan seguido ha tenido molestias debido a los siguientes problemas?**	**Nunca/Casi nunca**	**Algunas veces**	**A menudo**	**Siempre/Casi siempre**
1. ¿Ha pensado que no podía controlar su consumo de cannabis?				
2. ¿Le ha preocupado o puesto nervioso/a la posibilidad de que le pudiera faltar un porro?				
3. ¿Se ha sentido preocupado/a por su consumo de cannabis?				
4. ¿Ha deseado poder dejarlo?				

En los últimos 12 meses, ¿hasta qué punto le ha sido difícil dejar o estar sin consumir cannabis?

- Nada difícil
- Bastante difícil
- Muy difícil
- Imposible

A continuación vamos a preguntarle acerca del estado de su salud física.

¿Ha sufrido usted alguna de las siguientes enfermedades o estados de salud?

	SI	NO
Enfermedad del corazón, enfermedad coronaria, ataque al corazón		
Bronquitis crónica / Enfisema		
Infecciones respiratorias, tos, insuficiencia respiratoria		
Migraña (dolores de cabeza frecuentes)		
Problemas de sueño		
Enfermedad obstructiva pulmonar crónica (EPOC)		
Gastritis o úlcera		

Vómitos y diarrea		
Tumores/Cáncer (incluyendo cáncer en la sangre)		
Otros (especifique):		

DIFICULTAD RESPIRATORIA (DISNEA)

- En los últimos 30 días, ¿qué tanta dificultad tuvo para:
  - Respirar en reposo?
    - Ninguna
    - Poca
    - Moderada
    - Severa
    - Extrema
  - Respirar al hacer ejercicios leves, como subir 20 metros cuesta arriba o un tramo de escaleras (como 12 escalones)?
    - Ninguna
    - Poca
    - Moderada
    - Severa
    - Extrema
  - Debido a la tos o a silbidos en el pecho durante 10 minutos?
    - Ninguna
    - Poca
    - Moderada
    - Severa
    - Extrema

SUEÑO

- En una noche cualquiera, ¿cuántas horas duerme usualmente? ________
- En los últimos 30 días, ¿Qué tan a menudo tuvo problemas para quedarse dormido/a, porque se despertaba varias veces durante la noche, o porque se despertaba demasiado temprano por la mañana?
  - En ningún momento
  - Algunas veces
  - Una buena parte del tiempo
  - La mayoría del tiempo
  - Todo el tiempo

COMPRENSIÓN E INTERACCIÓN

- En los últimos 30 días, ¿qué tanta dificultad tuvo:
  - Para concentrarse o hacer algo durante 10 minutos?

        - Ninguna
        - Poca
        - Moderada
        - Severa
        - Extrema/No lo pudo hacer
    - Para recordar cosas importantes por hacer?
        - Ninguna
        - Poca
        - Moderada
        - Severa
        - Extrema/No lo pudo hacer
    - Para analizar y solucionar problemas en la vida diaria?
        - Ninguna
        - Poca
        - Moderada
        - Severa
        - Extrema/No lo pudo hacer
    - Para aprender algo nuevo, por ejemplo, aprender a llegar a un nuevo lugar?
        - Ninguna
        - Poca
        - Moderada
        - Severa
        - Extrema/No lo pudo hacer

DESTREZA Y ACTIVIDAD MOTORA FINA

- En los últimos 30 días, ¿qué tanta dificultad tuvo al usar sus manos y dedos, como por ejemplo para recoger pequeños objetos o para abrir o cerrar frascos?
    - Ninguna
    - Poca
    - Moderada
    - Severa
    - Extrema/No lo pudo hacer

SITUACIÓN LABORAL O ESCOLAR

Ahora me gustaría preguntarle acerca de las limitaciones que puede tener debido a su salud física y emocional, en el tipo de actividad que usted realiza, ya sea como empleado, trabajador independiente, estudiante o voluntario. Al responder me gustaría que pensara en los últimos 30 días.

- En los últimos 30 días, ¿qué tanta dificultad tuvo en:
    - Su trabajo diario?
        - Ninguna
        - Poca
        - Moderada
        - Severa

        - Extrema/No lo pudo hacer
    - Para terminar todo el trabajo que necesitaba hacer?
        - Ninguna
        - Poca
        - Moderada
        - Severa
        - Extrema/No lo pudo hacer
    - En los últimos 30 días, ¿cuántos días fue incapaz de ir a trabajar?
        - Registre el nº de días ________

A continuación le vamos a preguntar sobre las <u>lesiones o accidentes</u> que puede haber sufrido.

- ¿Ha sufrido algún accidente de tráfico?
    - Sí
    - No

¿Había consumido cannabis durante las 6 horas antes del accidente?

Sí

No

¿Fue usted considerado el/la responsable del accidente?

- Sí
- No

- ¿Ha vivido alguna de las siguientes situaciones en su núcleo familiar?
    - Enfados con violencia verbal (insultos, alzar la voz, etc.)
    - Enfados con violencia física
    - No
    - Otras, especificar
- ¿Quién ha ejercido violencia física?
    - Padre/madre sobre usted
    - Pareja sobre usted
    - Hijo/s sobre usted
    - Usted sobre su padre/madre
    - Usted sobre su pareja
    - Usted sobre sus hijo/s
    - Otras, especificar
- ¿Alguna vez ha pensado en hacerse daño a sí mismo/a?
    - Sí
    - No
- ¿Alguna vez los ha tratado de llevar a cabo?
    - Sí
    - No
